# A pan-cancer fingerprint: common molecular denominators of the human tumor microenvironment

**DOI:** 10.1038/s41392-021-00814-x

**Published:** 2021-11-11

**Authors:** Susanna S. Ng, Sonia Leonardelli, Michael Hölzel

**Affiliations:** Institute of Experimental Oncology, University Hospital Bonn, University of Bonn, 53127 Bonn, Germany

**Keywords:** Cancer, Immunology

In a recent study published in *Cancer Cell*, Bagaev et al. developed a pan-cancer tumor microenvironment (TME) classification that can be predictive of response to immune checkpoint inhibitor (ICI) therapy.^1^ The authors presented a detailed visualization that can be generated for each tumor, which combines the 29 functional gene expression signatures (Fges) used to subtype the TME with targetable genomic alterations.

Understanding the compartments and pathways within the TME could aid decision-making in patient-specific treatment of cancer, especially in the context of immunotherapies.^2^ The increasing use of ICIs have shown promising clinical outcomes in several cancer types. However, response to treatment is heterogenous and often results in immune-related adverse events.^3^ Therefore, efforts are focused on the development of prognostic tools to predict response to ICI.

**Fig. 1:**
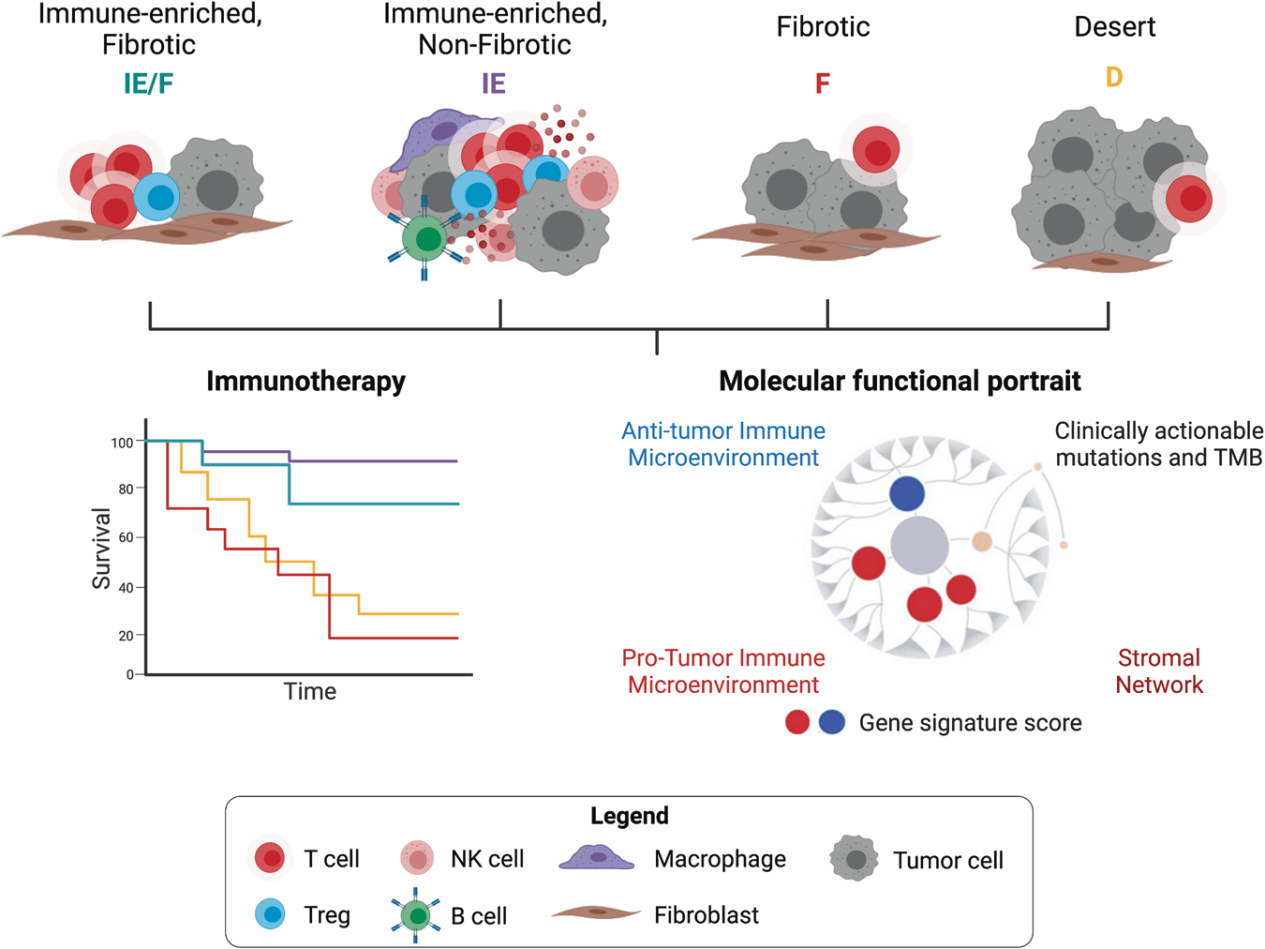
Four distinct pan-cancer tumor microenvironment (TME) subtypes. The schematic summarizes the features of the TME that define the immune-enriched, fibrotic (IE/F), immune-enriched, non-fibrotic (IE), fibrotic (F), and desert/immune-depleted (D) subtypes. These subtypes can predict immunotherapy response and survival outcome. The integration of subtype-defining gene expression signatures with data of genomic alterations generates a global visualization of tumors, termed the Molecular Functional (MF) portrait. TMB, tumor mutational burden. Figure generated on Biorender.com

Unsupervised analysis of 468 melanoma patients resulted in grouping of the TME into four major subsets based on manually curated Fges. These subtypes were defined by relative expressions of immune- and stromal-related signatures into (1) immune-enriched, fibrotic (IE/F); (2) immune-enriched, non-fibrotic (IE); (3) fibrotic (F); and (4) immune-depleted (D) (Fig. 1). Both immune-enriched subtypes expressed immune-related signatures and were distinguished by the expression of angiogenic and cancer-associated fibroblasts (CAF) activation signatures by the IE/F subset. Subtypes F and D conversely, were immune-depleted phenotypes containing higher tumor cell content relative to both immune-enriched subtypes. Notably, TMEs with an F subtype showed high expression of angiogenesis and CAFs, associated with expression of immune-suppressive transforming growth factor beta (TGF-β) and epithelial to mesenchymal transition, which was not observed in subtype D. TME subtype D was uniquely defined by an increased proliferation signature.

The application of these TME subtypes to transcriptomic data from 8042 samples of 24 different tumor entities within The Cancer Genome Atlas (TCGA) led to the identification of the same four TME clusters. This confirmed conservation of these TME subtypes on a pan-cancer level. The authors then investigated the prognostic value of the four TME subtypes and demonstrated that in most cancers, TMEs with an IE subtype had the most positive correlation with survival. Conversely, TMEs with an F subtype was associated with poor survival after accounting for cancer type and sex. However, while the prognostic potential of the four TME subtypes were shown for a variety of cancers and in multiple immunotherapy cohorts, the prognostic value and genomic features of the proposed subtypes could not be validated in some tumor types such as bladder carcinoma and cervical squamous cell carcinoma. Nevertheless, further comparison of the four TME subtype classification with two other well-known pan-cancer classification approaches—immunophenoscore and the six TCGA immune TME subtypes^4,5^—also indicated better predictive potential for overall survival (OS) in a variety of cancer entities including melanoma and gastro-esophageal adenocarcinoma.

Previously, predictive and prognostic biomarkers for ICIs have been generated by estimating the expression of immune checkpoint genes including PD-1, PD-L1, and CTLA-4 as well as assessing tumor mutational burden (TMB). However, in such cases, the heterogeneity of the TME was not considered. Earlier studies including the immunophenoscore and the six TCGA immune TME subtypes have incorporated genomic, transcriptomic, and immunological aspects of the TME. In comparison to these, the classification of TMEs into the four subtypes proposed in the study demonstrated greater predictability of response to immunotherapy. In addition, these subtypes also allowed prediction of response to other immune-based therapies, such as therapeutic vaccination and adoptive cell therapy. In both cases, response was positively correlated with the immune-enriched TME subtypes IE/F and IE.

Further analysis of the 8024 TCGA tumor types in the context of genomic alterations and their association with each TME subtype, showed no correlation between genomic aberrations and any of the TME clusters at a pan-cancer level, although this analysis provided an overview of clinically targetable mutations. The incorporation of the 29 Fges and clinically targetable mutations resulted in a Molecular Functional (MF) portrait that can be generated for each tumor. The authors proposed this as a tool that can inform treatment decisions in clinical settings. A limitation of bulk tumor transcriptomes is the absence of cell type-specific expression patterns and spatial context, which could be complemented by high-plex immunofluorescence technologies.

The use of the MF portrait provides a holistic evaluation of the TME. This facilitates better prediction of survival outcomes and response to therapy across different cancer types compared to existing biomarker panels. Retrospective evaluation of responders and non-responders to immunotherapy, as demonstrated in the publication, also allows a more in depth look into specific processes within the TME that are likely to contribute to patient outcome. This could be beneficial in clinical settings and for TME drug target identification.

While the depth of information represented by the MF portrait for each tumor provides a host of benefits, the implementation of processes and analyses required to generate such an encompassing view of each TME within the clinical environment presents some challenges. Within the publication, the information used for the development of the MF portrait that is subsequently proposed for use in rational therapeutic design is derived retrospectively from discovery-driven sequencing that are part of research projects such as the TCGA. Although the use of clinical genomics in oncology is on the rise, much of this is currently limited to specific panels that allow targeted sequencing of clinically approved drug targets and biomarkers.^2^ Extension of current clinical genomics practices into whole genome, exome, or transcriptome sequencing is required to obtain the information integrated in the MF portrait. However, challenges in time and financial costs, as well as data handling, analysis, and interpretation remain significant barriers to the routine implementation of prospective next-generation sequencing in clinical oncology outside of specialized centers. The development of extended array panels including genes that make up the 29 functional transcriptomic signatures presents a viable strategy to incorporate a pan-cancer signature for improved prediction of patient response and survival in clinical settings. Meanwhile, the use of MF portraits in retrospective research studies will provide greater insight into components and pathways of the TME that underlie responses to emerging immunotherapy candidates.
